# CXCR4 promotes B cell viability by the cooperation of nuclear factor (erythroid-derived 2)-like 2 and hypoxia-inducible factor-1α under hypoxic conditions

**DOI:** 10.1038/s41419-021-03615-w

**Published:** 2021-03-26

**Authors:** Ju-Won Jang, Pham Xuan Thuy, Jae-Wook Lee, Eun-Yi Moon

**Affiliations:** Department of Bioscience and Biotechnology, Sejong University, Seoul, 05006 Republic of Korea

**Keywords:** Stress signalling, Immune cell death

## Abstract

B cells that interact with T cells play a role in regulating the defense function by producing antibodies and inflammatory cytokines. C-X-C chemokine receptor type 4 (CXCR4) is a specific receptor for stromal cell-derived factor 1 (SDF-1) that controls various B cell functions. Here, we investigated whether CXCR4 regulates B cell viability by inducing hypoxia-inducible factor (HIF)-1α and nuclear factor (erythroid-derived 2)-like 2 (Nrf2) under a hypoxic condition in WiL2-NS human B cells. Nrf2 and CXCR4 expressions increased significantly when WiL2-NS cells were incubated under a hypoxic condition. Interfering with CXCR4 expression using CXCR4-siRNA inhibited cell viability. CXCR4 expression also decreased after treatment with a HIF inhibitor under the hypoxic condition, leading to inhibited cell viability. Increased reactive oxygen species (ROS) levels and the expression of HIF-1α and Nrf2 decreased under the hypoxic condition following incubation with N-acetylcysteine, a ROS scavenger, which was associated with a decrease in CXCR4 expression. CXCR4 expression was augmented by overexpressing Nrf2 after transfecting the pcDNA3.1-Nrf2 plasmid. CXCR4 expression decreased and HIF-1α accumulation decreased when Nrf2 was inhibited by doxycycline in tet-shNrf2-expressed stable cells. Nrf2 or HIF-1α bound from −718 to −561 of the CXCR4 gene promoter as judged by a chromatin immunoprecipitation assay. Taken together, these data show that B cell viability under a hypoxic condition could be regulated by CXCR4 expression through binding of HIF-1α and Nrf2 to the CXCR4 gene promoter cooperatively. These results suggest that CXCR4 could be an additional therapeutic target to control B cells with roles at disease sites under hypoxic conditions.

## Introduction

B cells affect tumor development and behavior through pro-tumor and anti-tumor immune responses^1,2^. B cells produce antibodies and release various cytokines^3–5^ to modulate immune responses^6,7^. B cells are exposed to a variety of oxygen concentrations that determine their migration, development, and differentiation^8^. Oxygen concentration is highly associated with the division, proliferation, and survival of cells^9–14^. Low oxygen tension is related to a variety of pathological conditions, including cancer, rheumatoid arthritis, chronic inflammatory bowel disease, and ischemia/reperfusion injury^15^. Hypoxia is a feature of physiological and pathological immunological niches^10^. Under hypoxic conditions, many immune cells, including B cells, play a role in controlling the disease condition^8^. However, little has been reported about how B cells are controlled under hypoxic conditions.

Hypoxia and the hypoxia-inducible factor (HIF) signaling pathway are crucial for B cell development and function, such as survival, proliferation, and cytokine production^16^. Inappropriate regulation of B cells contributes to various diseases, including autoimmune, malignant, allergic, and other conditions^6,17^*.* Therefore, B cell behavior should be studied under hypoxic conditions to understand the regulation of B cell-associated diseases^18,19^. However, little is known about which factors play a role in regulating B cells under hypoxic conditions.

HIF is a heterodimeric transcription factor comprising α and β subunits in the mammalian response to low oxygen level^20^. The two prolines on HIF-1α are hydroxylated by prolyl hydroxylase (PHD) and the HIF-1α protein is degraded by ubiquitination with the von Hippel–Lindau (VHL) complex under normoxic conditions^21^. Under hypoxic conditions, HIF-1α is stabilized, dimerized with the HIF-1β subunit, and binds to hypoxia response elements in the nucleus^22^. A hypoxic condition also increases the production of reactive oxygen species (ROS) to activate HIF1-α by inactivating PHD^23,24^. Hypoxia-driven ROS activates nuclear factor erythroid 2-like 2 (Nrf2), which plays a crucial role in regulating the transcription of antioxidant genes to reduce ROS accumulation^25^.

Nrf2 is negatively regulated by binding to the Kelch-like erythroid cell-derived protein with cap’n’collar homology-associated protein 1 and proteasomal degradation via Cullin3^26^. Then, Nrf2 translocates to the nucleus and subsequently binds to antioxidant response elements (ARE) on promoters of antioxidant genes, such as NADPH and heme oxygenase 1^27^. The Nrf2 protein is closely related to decreased survival and increased metastasis in several cell types^28^. B cell survival is associated with oxidative stress-mediated activation of Nrf2^29^.

Chemokine receptors cause cells to migrate toward a chemotactic cytokine gradient (chemotaxis). Among them, CXCR4 is a 352 amino acid rhodopsin-like G protein-coupled receptor that selectively binds CXC chemokine stromal cell-derived factor 1 (SDF-1) known as CXCL12^30,31^. CXCR4 is selectively induced by activating HIF-1 on different types of cancer cells under hypoxic conditions^32–35^. The von Hippel–Lindau tumor suppressor protein pVHL negatively regulates CXCR4 expression by targeting HIF for degradation under normoxic conditions^36^. In addition, CXCR4 is expressed on monocytes, B cells, and naive T cells^37,38^. CXCR4 is produced by all subsets of B cells and plays a role in the homeostasis of B cell compartments and humoral immunity^39,40^. Little is known about how CXCR4 expression is regulated by Nrf2 and HIF-1α to control B cell survival under hypoxic conditions.

In this study, we investigated whether B cell viability was regulated by CXCR4 via the cooperation of Nrf2 and HIF-1α under hypoxic conditions. Our data showed that CXCR4 expression was regulated by HIF-1α and Nrf2 in response to hypoxia-induced ROS, leading to the regulation of B cell survival. These results suggest that CXCR4 could be a novel therapeutic target to control B cells under hypoxic conditions.

## Materials and methods

### Reagents

Hygromycin B and doxycycline were purchased from Cayman (Ann Arbor, MI, USA). N-acetyl-l-cysteine (NAC) and antibodies to β-tubulin were purchased from Sigma-Aldrich (St. Louis, MO, USA). HIF- inhibitor (sc-205346) and antibodies to HIF-1α (sc-10790) or Nrf2 (sc-365949) were obtained from Santa Cruz Biotechnology (CA, USA). Antibodies to CXCR4 (35-8800) were obtained from Invitrogen, Thermo Fisher Scientific. (Waltham, MA, USA). 2′,7′–dichlorofluorescin diacetate (DCF-DA) was purchased from Molecular Probe (Eugene, OR, USA). CellTiter-Glo substrates and ViaFect™ were purchased from Promega Co. (Madison, WI, USA). pCDNA3.1-Nrf2 plasmids were kindly provided from Professor Byung-Chul Kim, Division of Life Sciences, Kangwon National University, Chuncheon, Republic of Korea. Except indicated, all chemicals were obtained from Sigma-Aldrich (St. Louis, MO, USA)^41^.

### Cell cultures

WiL2-NS, a human B lymphoblast cells was acquired from the Korea Research Institute of Bioscience and Biotechnology (KRIBB) cell bank (Daejeon, Korea). Cells were tested if they were free from mycoplasma contamination. Cells were incubated with RPMI medium 1640 (GIBCO, Grand Island, NY, USA) supplemented with 10% heat-inactivated fetal bovine serum (FBS) (GIBCO, Grand Island, NY, USA), 100 units/ml of penicillin/streptomycin and 2 mM l-glutamine (GIBCO, Grand Island, NY, USA) at 37 °C humidified incubator with 5% CO_2_ condition^41,42^.

### Hypoxia treatment^43^

For incubation under hypoxic condition, cells were placed in an atmosphere of 1% O_2_, 5% CO_2_, 10% H_2_, and 84% N_2_ with intermittent flushing with nitrogen, sealed, and then maintained in a humidified incubator at 37 °C in a hypoxic chamber (Forma Anaerobic System, Thermo Electron Corporation, Marietts, OH, USA). Hypoxia-treated cells were harvested inside hypoxic chamber to prevent the rapid degradation of hypoxia-responsive molecules.

### Preparation of the stable Nrf2-knockdown (KD) cells^41^

Lentiviral vector of Nrf2-shRNA (shNrf2) was packaged into virus particle by the method reported previously^44^, which was provided by Sang-Min Jeon, Professor, College of Pharmacy, Ajou University, Gyeonggido, Republic of Korea. 293T cells were transfected with lentiviral vector using Lipofectamine^®^ 2000 according to the recommended protocol on the Addgene website. Lentivirus-containing conditioned medium (LCCM) was aliquoted into 1 ml stock in each cryovial. Then, WiL2-NS stable cells that do not express Nrf2 (tet-shNrf2, +Dox) were prepared as follows. Briefly, WiL2-NS 1 × 10^5^ cells were incubated in each well of six-well plate overnight. Cell culture in 500 μl medium of each well was mixed with 1 ml LCCM and 1.2 μl polybrene (Millipore TR-1003-G). Culture medium was changed with 2 ml fresh medium containing 250 μg/ml hygromycin (Cayman 14291). The infected shNrf2-positive control cells (tet-shNrf2, -Dox) were selected by the treatment with hygromycin B (250 µg/ml) every 3 days. Nrf2-KD cells were obtained and maintained by the treatment with 0.2 μg/ml doxycycline (Cayman 14422) every 2 days.

### Cytotoxicity assay

Cell survival was quantified by counting cells with trypan blue assay^41^. For trypan blue exclusion assay, cell suspension was mixed with equal volume of 0.4% trypan blue in PBS. Dying or dead cells were stained with blue color and viable cells were unstained. Each cell was counted by using hemocytometer under light microscope (Olympus Korea Co., Ltd, Seoul, Republic of Korea). Total cell number in each state was calculated by the multiplication with dilution factor.

### Measurement of ROS^41^

Intracellular ROS level was determined by incubating cells with or without 10 μM of 2′,7′-dichlorofluorescin diacetate (DCF-DA) (Molecular Probe, Eugene, USA) at 37 °C for 20 min. Fluorescence intensity of 10,000 cells was analyzed by CELLQuest™ analyzing software in FACS Calibur™ (Becton Dickinson, San Joes, CA, USA). Also, intracellular ROS level was observed with DCF-DA by fluorescence microscopy.

### Transfection of nucleic acids^45^

Each plasmid DNA, siRNAs for CXCR4 and AccuTarget™ negative contol siRNA were transfected into cells as follows. Briefly, each nucleic acid and Viafect™ (Promega Co., Madison, USA) was diluted in serum-free medium and incubated for 5 min, respectively. The diluted nucleic acid and Viafect™ reagent was mixed by inverting and incubated for 20 min to form complexes. In the meanwhile, cells were stabilized by the incubation with culture medium without antibiotics and serum for at least 2 h prior to the transfection. Pre-formed complexes were added directly to the cells and cells were incubated for an additional 6 h. Then, culture medium was replaced with antibiotic and 10% FBS-containing DMEM and incubated for 24–72 h prior to each experiment.

Nrf2 was overexpressed by the transfection of cells with pCDNA-Nrf2 plasmid DNA, which was accompanied with pCDNA3.1 for control group using Viafect™.

### Gaussia luciferase assay^45^

Pre-designed promoters for hCXCR4 (NM_003467) were obtained from GeneCopoeia Inc. (Rockville, MD, USA). hCXCR4-promoter (HPRM44928) was 1309 bp (−1328 to −35) upstream from starting codon for hCXCR4 transcription in *Homo sapiens* BAC RP11-809C23 from chromosome 2 (AC068492.2). Schematic figures and sequences of promoters were shown in Supplementary Fig. S1. CXCR4 mutant promoter 66 bp (−100 to −35) was prepared by the deletion of wildtype upstream. Wildtype and mutant promoters were cloned into Gaussia luciferase (Gluc) reporter plasmid vector, pEZX-PG02.

WiL2-NS cell were transfected with wildtype or mutant pEZX-PG02-hCXCR4-Gluc plasmid DNA using ViaFect™ (Promega Co., Madison, WI, USA) to measure the activity of hCXCR4 promoter. At the same time, cells were co-transfected with pcDNA-lacZ for monitoring transfection efficiency by β-galactosidase assay. Then, cells were incubated for an appropriate time. Secreted Gluc reporter protein was obtained by the collection of culture-conditioned media after the indicated time intervals. Gluc activity of reporter protein was measured by BioLux^®^ Gluc assay kit (New England BioLabs, Ipswich, MA, USA) including coelenterazine as a substrate for Gluc according to the manufacturer’s protocol. Luminescence was measured using luminometer (Berthold Technologies, Oak Ridge, TN, USA). Luciferase activity unit was normalized to this control β-galactosidase activity.

### Reverse transcriptase polymerase chain reaction (RT-PCR)

Total of RNA was extracted from WiL2-NS cells by Nucleozol (BMS). 1 µg of total RNA was used for synthesizing cDNA. With using Oligo (dT) 18 mer primer (macrogen) and superscript reverse transcriptase (Bioneer) were added with follows: PCR amplification was performed to detect hCXCR4 (using primer forward; 5′-CAG TGA GGC AGA TGA CAG AT-3′, reverse; 5′-CAG GAC AGG ATG ACA ATA CCA-3′), and β-actin (using primer forward; 5′-GTC ACC AAC TGG GAC GAC AT-3′, reverse; 5′-GCA CAG CCT GGA TAG CAA CG-3′).

### Realtime quantitative PCR analysis (qPCR)

To perform realtime quantitative PCR (qPCR), total cellular RNA (5 μg) was reverse transcribed into cDNA as described in RT-PCR. Real-time qPCR was performed using the CFX96 Touch™ Real-Time PCR Detection System (Bio-Rad laboratories, Hercules, CA, USA). The RT reaction product (100 ng) was amplified with Thunderbird™ SYBR qPCR mix (TOYOBO Co. Ltd., Osaka, Japan) using primers specific for target genes, hCXCR4 primers (forward; 5′-CAG TGA GGC AGA TGA CAG AT-3′, reverse; 5′-CAG GAC AGG ATG ACA ATA CCA-3′), and β-actin primers (forward; 5′-GCC AGG TCA TCA CCA TTG-3′, reverse; 5′-GTT GAA GGT AGT TTC GTG GAT-3′). Samples were heated to 95 °C for 1 min and amplified for 40 cycles (95 °C for 10 s, 55 °C for 10 s and 72 °C for 30 s) followed by a final extension step of 72 °C for 10 min. β-actin was used as an internal control. Relative quantification of each mRNA was analyzed by the comparative threshold cycle (CT) method and normalized to β-actin expression using Bio-Rad CFX Manager™ Software^43^.

### Chromatin immunoprecipitation (ChIP) assay

ChIP assay were performed as describied previously^43,46,47^. Cells were crosslinked with final concentration 1% formaldehyde for 10 min at room temperature. Then, 125 mM glycine was added to quench unreacted formaldehyde. Cells were gathered and sonicated to make DNA fragments with a size range of 200–1000 bp. Cell extracts were immune-precipitated using 2 μg anti-Nrf2, anti-HIF-1α, or rabbit IgG control (Abcam, Cambridge, UK) for each sample suspended in 450 μl ChIP dilution buffer (0.01% SDS, 1.1% Triton X-100, 1.2 mM EDTA, 16.7 mM Tris–HCl, pH 8.1, 167 mM NaCl) purchased from Cell signaling Technology (Cat# 20-153, Danvers, MA). For all ChIP experiments, PCR analysis were performed by using multiple sets of primers spanning the transcription factor-binding site on hCXCR4 gene promoter.

### Western blot analysis^43^

Cellular proteins were lysed by 0.5% Nonidet P-40 (NP-40) lysis buffer containing 150 mM NaCl, 20 mM Tris–HCl (pH 8.2), protease inhibitor (2 µg/ml aprotinin, 2 µg/ml pepstatin, 1 mM phenylmethylsulfonyl fluoride, 1 µg/ml leupeptin) and phosphatase inhibitor (5 mM sodium fluoride and 1 mM sodium vanadate). Cells were lysed for 30 min on ice and centrifuged at 12,000×*g* for 20 min at 4 °C. Protein concentrations of lysates were determined by using SMART TM BCA protein assay kit (Pierce 23228). Equal sum of cellular proteins in sodium dodecyl sulfate (SDS) sample buffer were denatured by boiling at 100 °C for 5 min.

Samples were separated with protein size by SDS–polyacrylamide gel electrophoresis (SDS–PAGE). Separated samples of SDS–PAGE were transferred to nitrocellulose membrane. Membranes were blocked with 2% gelatin or 5% non-fat skim milk in Tris-buffered saline containing 0.05% Tween 20 (TBST). After blocking, the primary antibodies reactive specific targets were incubated for several hours. Secondary antibodies (horse radish peroxidase (HRP)-conjugated) were used to visualize target-specific primary antibody by reaction with D-Plus™ chemi-luminescence (ECL) system (Dongin Life Science, Seoul, Republic of Korea). Immuno-reactive targets were detected by X-ray films (Agfa healthCare, CP-BU new).

### Statistical analysis system

Experimental differences were determined separately for statistical significance using Students’ *t*-test and ANOVA. The *p* value of *p* < 0.05 (*, ^#^, ^&^ or ^$^), *p* < 0.01 (**, ^##^, ^&&^ or ^$$^) was judged to be significant.

## Results

### hCXCR4 expression regulates B cell viability under hypoxic conditions

**Fig. 1:**
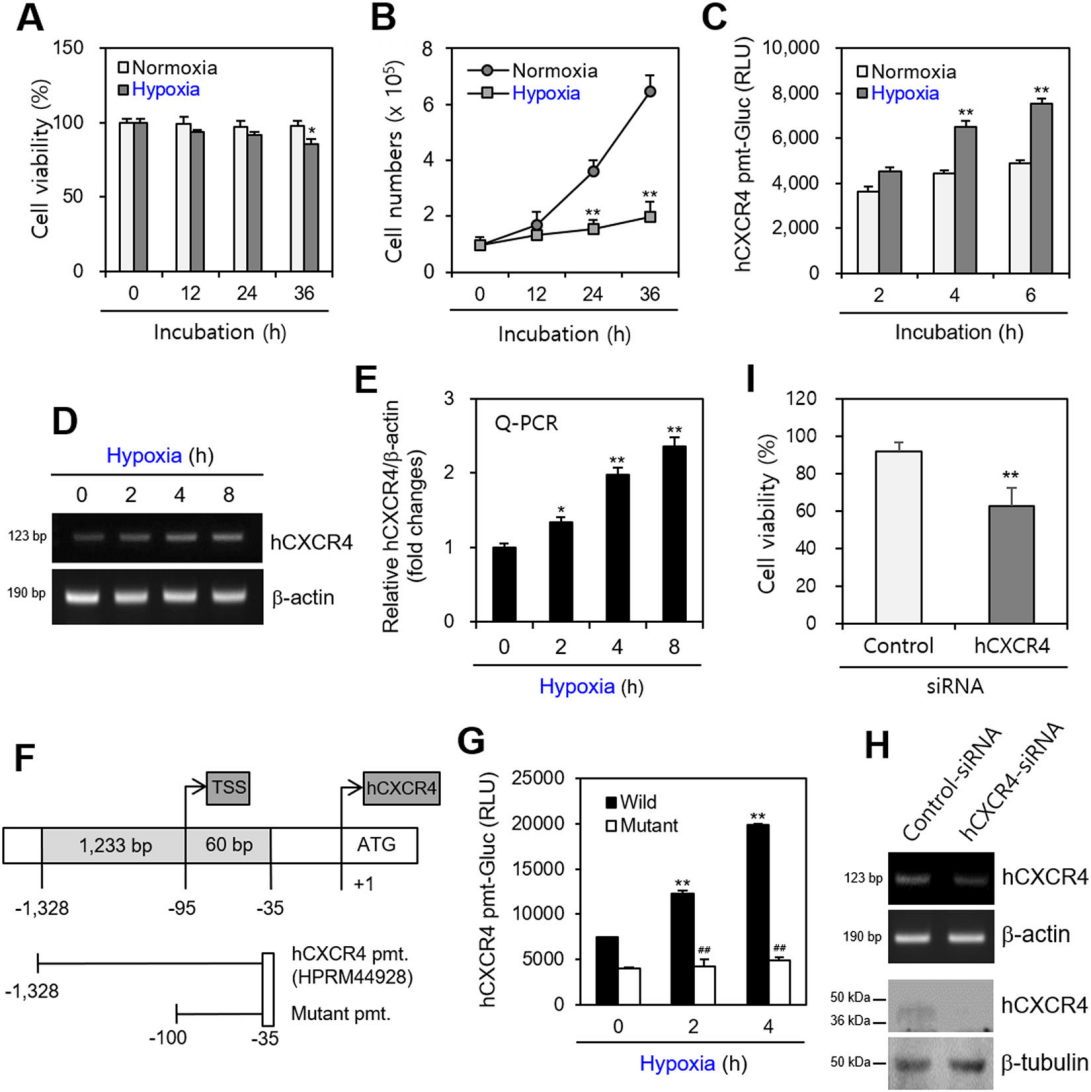
B cell viability is regulated by hCXCR4 expression under hypoxic condition. **A**, **B** WiL2-NS cells were incubated for 12, 24, 36 h under normoxic or hypoxic conditions. Cell viability was assessed by using trypan blue exclusion assay (**A**) and cell number was calculated (**B**). **C**–**E** WiL2-NS cells were transfected with pEZX-PG02-hCXCR4-gaussia luciferase (Gluc) plasmid DNA using Viafect™. After 30 h transfection, WiL2-NS cells were incubated with hypoxia and Gluc activity of hCXCR4 promoter (pmt) was measured by using luminometer (**C**). RNA was extracted by using Nucleozol. mRNA of hCXCR4 was measured by RT-PCR (**D**) or real-time qPCR (**E**). **F**, **G** Mutant promoter to hCXCR4 was prepared from wild type promoter (**F**). WiL2-NS cells were transfected with wild-type or mutant-type pEZx-PG02-hCXCR4-Gluc promoter plasmid DNA. 30 h after transfection, WiL2-NS cells were incubated under hypoxic condition and Gluc activity was measured by using luminometer (**G**). **H**, **I** Cells were transfected with CXCR4-siRNA under normoxic condition. RNA was extracted by using Nucleozol. mRNA of hCXCR4 was measured by RT-PCR (**H**, top). Cell lysates were prepared and the level of each protein was measured by western blot analysis (**H**, bottom). Cell viability under hypoxic conditions was assessed by trypan blue exclusion assay (**I**). Each experiment was performed at least five times. Data in bar or line graphs stands for the means ± SD. **p < 0.01; significantly different from control group under normoxic condition (**A**–**C**, **E**, **G**) or negative control siRNA-treated group (**H**). ^##^*p* < 0.01; significantly different from control transfected with wildtype hCXCR4-pmt at each time point (**G**).

Given that hCXCR4 is expressed^37,38^ and B cells play a role in controlling the disease condition^8^ under hypoxic conditions, we examined whether hCXCR4 expression controlled B cell viability. To test the effect of hypoxia on B cells, WiL2-NS cells were incubated under normoxic and hypoxic conditions. Cell viability and total cell number decreased after incubation under hypoxic conditions compared with that under normoxic conditions as judged by the trypan blue exclusion assay (Fig. 1A and B). In response to 36 h incubation, cell viability under hypoxic condition is ~12% lower than that under normoxic (Fig. 1A) and an increase in total cell number was twice under hypoxic condition compared to that 6.5 times under normoxic condition (Fig. 1B). When WiL2-NS cells were transfected with pEZX-PG02-hCXCR4-gaussia luciferase (Gluc) plasmid DNA and incubated under hypoxic conditions, hCXCR4 promoter activity increased ~20%, ~30%, or ~35% after 2, 4, or 8 h of incubation under hypoxic conditions (Fig. 1C). These results were confirmed by reverse transcription-polymerase chain reaction (RT-PCR) and qPCR analyses (Fig. 1D and E). hCXCR4 expression increased ~1.2, ~2.0, and ~2.4 times after 2-, 4-, and 8-h incubations, respectively, under hypoxic conditions (Fig. 1E). To confirm the effect of hypoxia on hCXCR4 expression, we prepared a deletion mutant type (−100 to −35) of the rhCXCR4 promoter (Fig. 1F). When WiL2-NS cells were transfected with wild-type and mutant-type pEZX-PG02-hCXCR4-Gluc plasmid DNA and incubated under hypoxic conditions, Gluc activity decreased ~45%, ~60%, and ~75% after 2-, 4-, and 8-h incubations under hypoxic conditions compared to those under normoxic conditions after deleting upstream of the hCXCR4 promoter (Fig. 1G). When hCXCR4 expression was inhibited by transfection with hCXCR4–siRNA as judged by RT-PCR (Fig. 1H, top) and western blot analysis under normoxic conditions (Fig. 1H, bottom), cell viability under hypoxic conditions decreased ~33% compared to that treated with negative control siRNA (Fig. 1I), suggesting that B cell viability is regulated by hCXCR4 expression under hypoxic conditions.

### Hypoxia-induced HIF-1α regulates Nrf2 and hCXCR4 expression

**Fig. 2:**
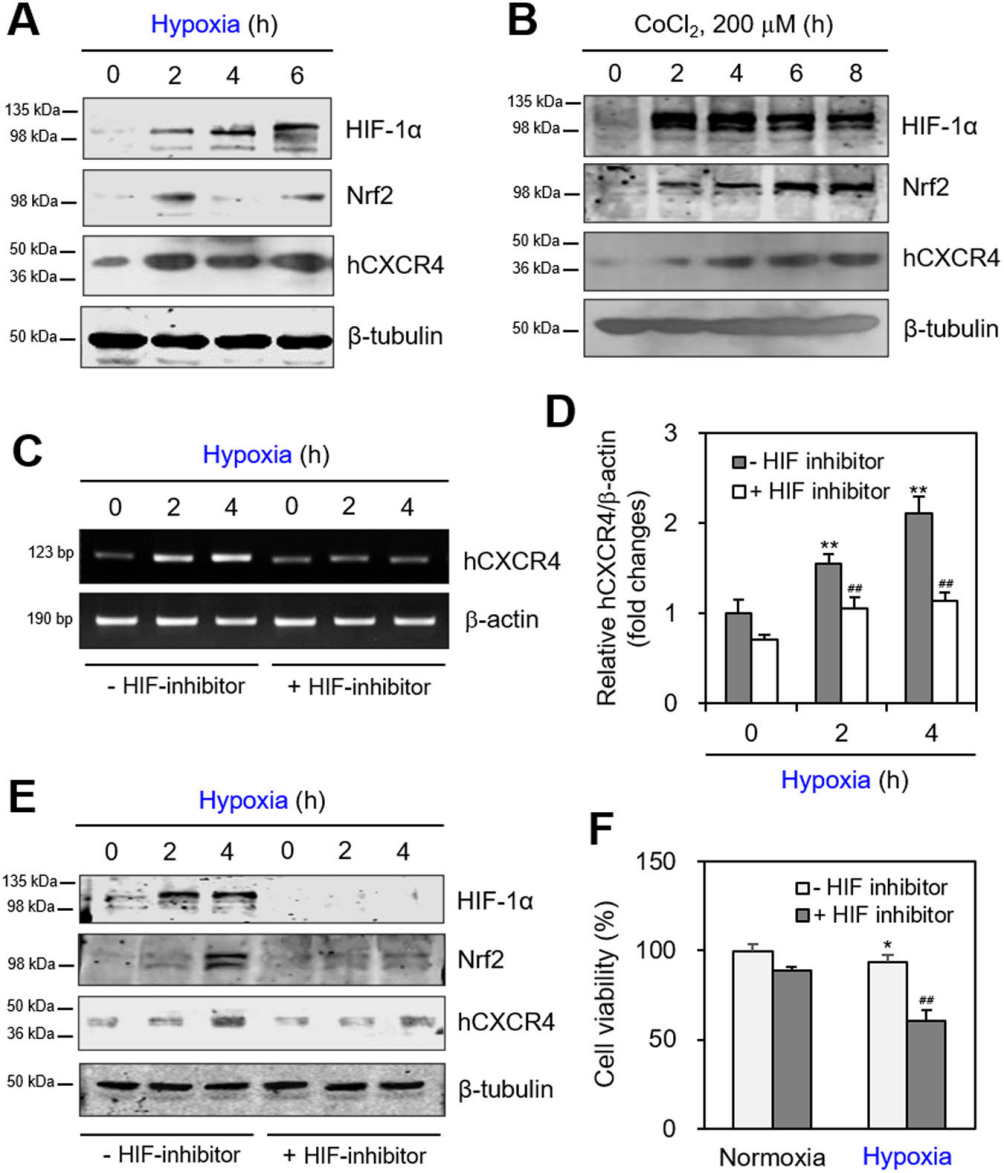
hCXCR4 expression was reduced by the inhibition of HIF-1α. **A**, **B** WiL2-NS cells were incubated under hypoxic condition (**A**) or with CoCl_2_ treatment (**B**) for up to 8 h. Cell lysates were prepared and the level of each protein was measured by western blot analysis. **C**–**F** WiL2-NS cells were incubated in the presence or the absence of HIF inhibitor under hypoxic conditions. RNA was extracted by using Nucleozol. mRNA of hCXCR4 was measured by RT-PCR (**C**) or qPCR (**D**). Relative hCXCR4 transcripts were represented as bar graph (**D**). Cell lysates were prepared and the level of each protein was measured by western blot analysis (**E**). Cell viability was assessed by trypan blue exclusion assay and represented as bar graph (**F**). Each experiment was performed at least five times. Data in bar graphs represented the means ± SD. *p < 0.05, **p < 0.01; significantly different from control group under normoxic condition. ^#^*p* < 0.05, ^##^*p* < 0.01; significantly different from HIF-1α inhibitor-untreated group (**D**, **F**).

As hCXCR4 is regulated by hypoxia-induced HIF-1α^38^, we investigated whether hypoxic conditions regulate hCXCR4 expression in B cells. Thus, WiL2-NS cells were incubated under hypoxic conditions or with CoCl_2_. As shown in Fig. 2A, the hypoxic conditions enhanced HIF-1α, Nrf2, and hCXCR4 as judged by western blot analysis. Enhancement of these molecules was also confirmed by treatment with the chemical hypoxic mimicker CoCl_2_ (Fig. 2B). Hypoxic condition is not the same to chemical hypoxia induced by CoCl_2_ stabilizing HIF-1α. So, Nrf2 expression pattern in response to CoCl_2_ is more long-lasting than that by hypoxia. Increased hCXCR4 expression was attenuated by co-treatment with the HIF inhibitor according to the RT-PCR analysis (Fig. 2C) and was confirmed by qPCR. The HIF inhibitor inhibited hCXCR4 expression by ~35% and ~45% after 2- and 4-h incubations under hypoxic conditions, respectively (Fig. 2D), which was confirmed by western blot analysis (Fig. 2E). Changes in cell viability in response to the HIF inhibitor were assessed by the trypan blue exclusion assay. The HIF inhibitor reduced B cell viability ~33% under hypoxic conditions (Fig. 2F). These results suggest that hCXCR4 could enhance B cell viability and might be associated with the expression of Nrf2 and HIF-1α under hypoxic conditions.

### Hypoxia-induced ROS affect HIF-1α, Nrf2, and hCXCR4 expressions

**Fig. 3:**
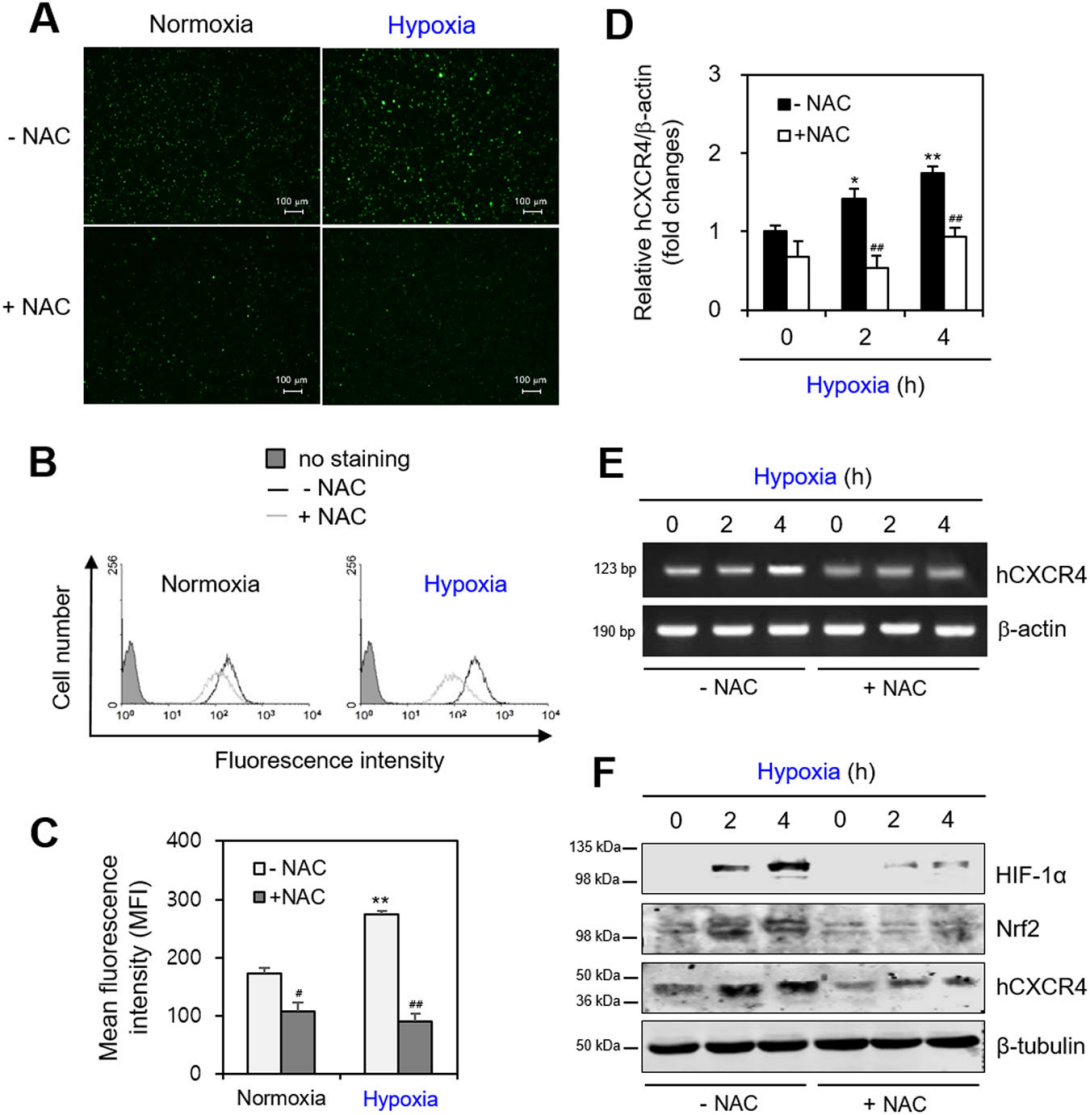
CXCR4 expression was inhibited by the treatment with N-acetyl-l-cysteine (NAC). **A**–**F** WiL2-NS cells were incubated in the presence or absence of N-acetyl-l-cysteine (NAC) under hypoxic condition and treated with DCF–DA. ROS production was observed under fluorescence microscope (**A**) or it was measured by flow cytometry analysis (**B**). The mean fluorescence intensity (MFI) was analyzed by WinMDI 2.8 for ROS level and represented as bar graph (**C**). RNA was extracted by using Nucleozol. mRNA of hCXCR4 was measured by qPCR (**D**) or RT-PCR (**E**). Cell lysates were prepared and the protein level of each protein was measured by using western blot analysis (**F**). Each experiment was performed at least five times. Data in a bar graph represented the means ± SD. *p < 0.05, **p < 0.01; significantly different from control group under normoxic condition. ^#^*p* < 0.05, ^##^*p* < 0.01; significantly different from NAC-untreated group (**C**, **D**).

As hypoxia increases ROS^24^, we determined whether hypoxia changed intracellular ROS levels in B cells using DCF-DA. When WiL2-NS cells were incubated in the presence or absence of NAC under hypoxic conditions, ROS production was observed under a fluorescence microscope (Fig. 3A, top). ROS production was also measured by flow cytometry analysis (Fig. 3B, left), which could show the distribution of each single cell having different level of ROS. Then, results revealed that the mean fluorescence intensity (MFI) under the hypoxic condition was higher than that under a normoxic condition (Fig. 3C, light gray bar). ROS production decreased in response to NAC, which was assessed by a decrease in the number of fluorescent cells (Fig. 3A, bottom), a left-ward shift in the histogram (Fig. 3B, right), and reduced MFI (Fig. 3C, dark gray bar). hCXCR4 expression under the hypoxic condition was also attenuated by the NAC treatment. hCXCR4 expression was measured by qPCR, which showed that NAC inhibited hCXCR4 expression ~60% and ~40% after 2- and 4-h incubations under hypoxic conditions, respectively (Fig. 3D). The reduced hCXCR4 expression in response to NAC was confirmed by RT-PCR (Fig. 3E) and western blot analyses (Fig. 3F). Nrf2 level in the absence of NAC was consistent with the previous result (Fig. 2A) by the incubation for 2 h under hypoxic condition. The protein levels of HIF-1α and Nrf2 were inhibited by NAC under hypoxic conditions (Fig. 3F). These results suggest that hypoxia-induced ROS regulate hCXCR4 expression.

### Hypoxia-induced Nrf2 regulates HIF-1α and hCXCR4 expressions

**Fig. 4:**
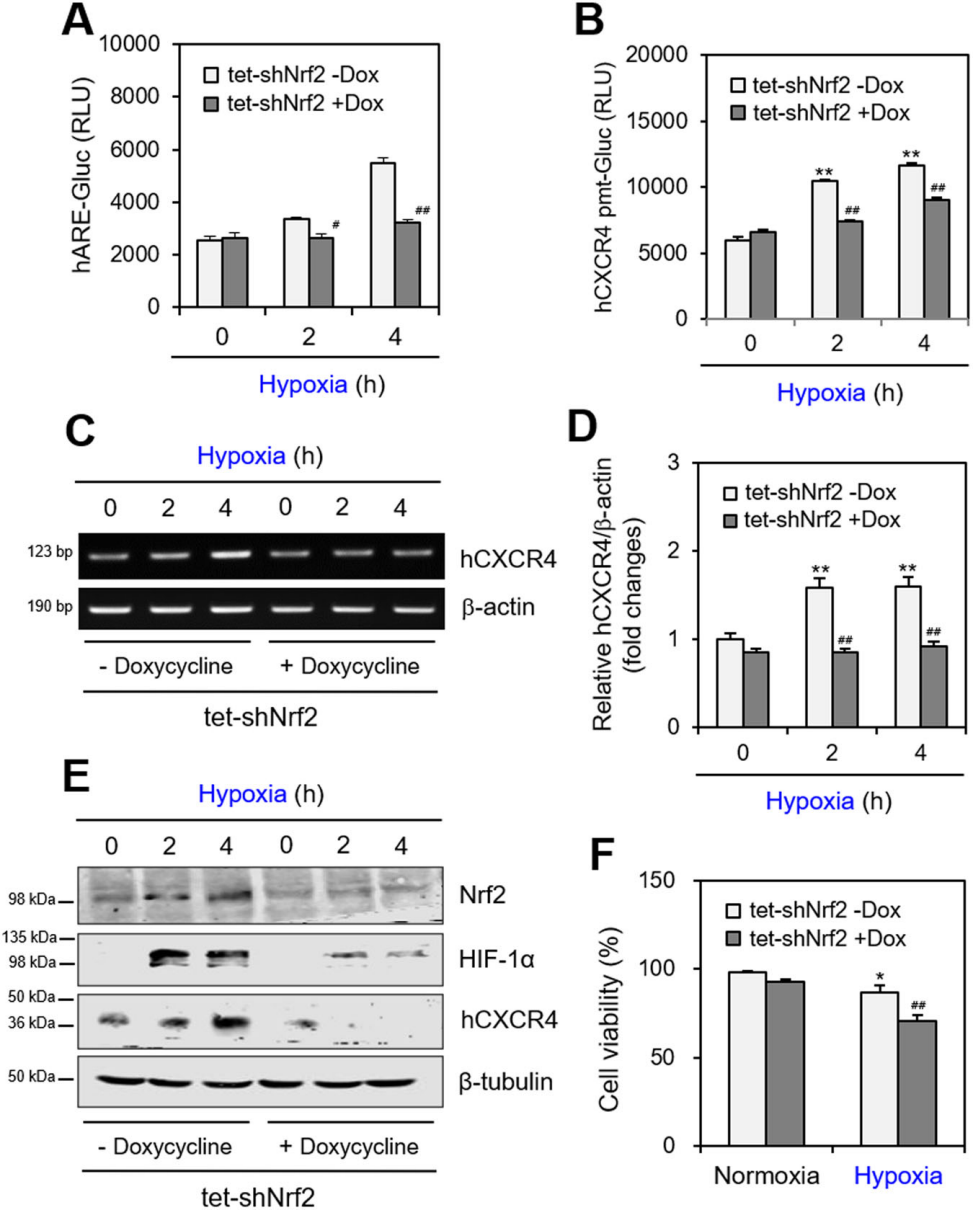
hCXCR4 expression was attenuated by tetracycline (tet)-inducible Nrf2-knockdown. **A**, **B** Nrf2-knockdown (KD) cells were prepared by the infection with lentivirus-packaged shNrf2, colony selection and the treatment with 0.2 μg/ml doxycycline (Dox) for 48 h. Then, the infected shNrf2-positive control (tet-shNrf2, −Dox) and Nrf2-KD (tet-shNrf2, +Dox) cells were transfected with pEZx-PG02-hARE-Gluc plasmid DNA (**A**) or pEZx-PG02-hCXCR4-Gluc promoter (pmt) (**B**). 30 h after transfection, cells were incubated under hypoxic condition and Gluc activity of hARE (**A**) or hCXCR4-pmt (**B**) was measured by using luminometer. **C**–**F** The infected shNrf2-positive control (tet-shNrf2, −Dox) and Nrf2-KD (tet-shNrf2, +Dox) WiL2-NS cells were incubated under normoxic or hypoxic conditions. RNA was extracted by using Nucleozol. mRNA of hCXCR4 was measured by qPCR (**C**) or RT-PCR (**D**). Cell lysates were prepared and the protein level of each protein was measured by using western blot analysis (**E**). Cell viability was measured by using trypan blue exclusion assay (**F**). Each experiment was performed at least five times. Data in bar graphs represented the means ± SD. *p < 0.05, **p < 0.01; significantly different from control group under normoxic condition. ^#^*p* < 0.05, ^##^*p* < 0.01; significantly different from shNrf2-positive control (tet-shNrf2, −Dox) at each time point (**A**–**C**, **F**).

To investigate whether hypoxia-induced Nrf2 regulates hCXCR4 expression, we prepared tetracycline (tet)-inducible Nrf2-knockdown (KD) cells by infecting with lentivirus-packaged shNrf2 and treating with doxorubicin (Dox). Nrf2-KD was confirmed by transfection with human antioxidant response element (hARE)-Gluc plasmid DNA. hARE-Gluc activity in Nrf2-KD (tet-shNrf2, +Dox) WiL2-NS cells was attenuated ~20% and ~40% after 2- and 4-h incubations under hypoxic conditions, respectively, compared to those in shNrf2-positive control cells (tet-shNrf2, −Dox) (Fig. 4A). hCXCR4 expression decreased in Nrf2-KD (tet-shNrf2, +Dox) WiL2-NS cells under hypoxic conditions. The inhibitory effect of Nrf2 on hCXCR4 expression was confirmed under hypoxic conditions by measuring hCXCR4-Gluc activity and expression by qPCR. hCXCR4-Gluc activity in Nrf2-KD cells decreased ~33% and ~25% after 2- and 4-h incubations under hypoxic conditions, respectively, compared to those in shNrf2-positive control cells (Fig. 4B). hCXCR4 expression in Nrf2-KD cells decreased ~50% and ~45% after 2- and 4-h incubations under hypoxic conditions, respectively, compared to those in shNrf2-positive control cells (Fig. 4C). The qPCR data were confirmed by RT-PCR (Fig. 4D). Western blot analysis revealed that HIF-1α and hCXCR4 expression decreased in response to Nrf2-KD under hypoxic conditions (Fig. 4E). When cells were incubated under hypoxic conditions, the viability of Nrf2-KD cells decreased by 20% compared to that in shNrf2-positive control cells (tet-shNrf2, −Dox) (Fig. 4F). These results suggest that Nrf2 regulates HIF-1α and hCXCR4 expressions under hypoxic conditions.

### Nrf2 and HIF-1α regulate hCXCR4 expression and B cell viability

**Fig. 5:**
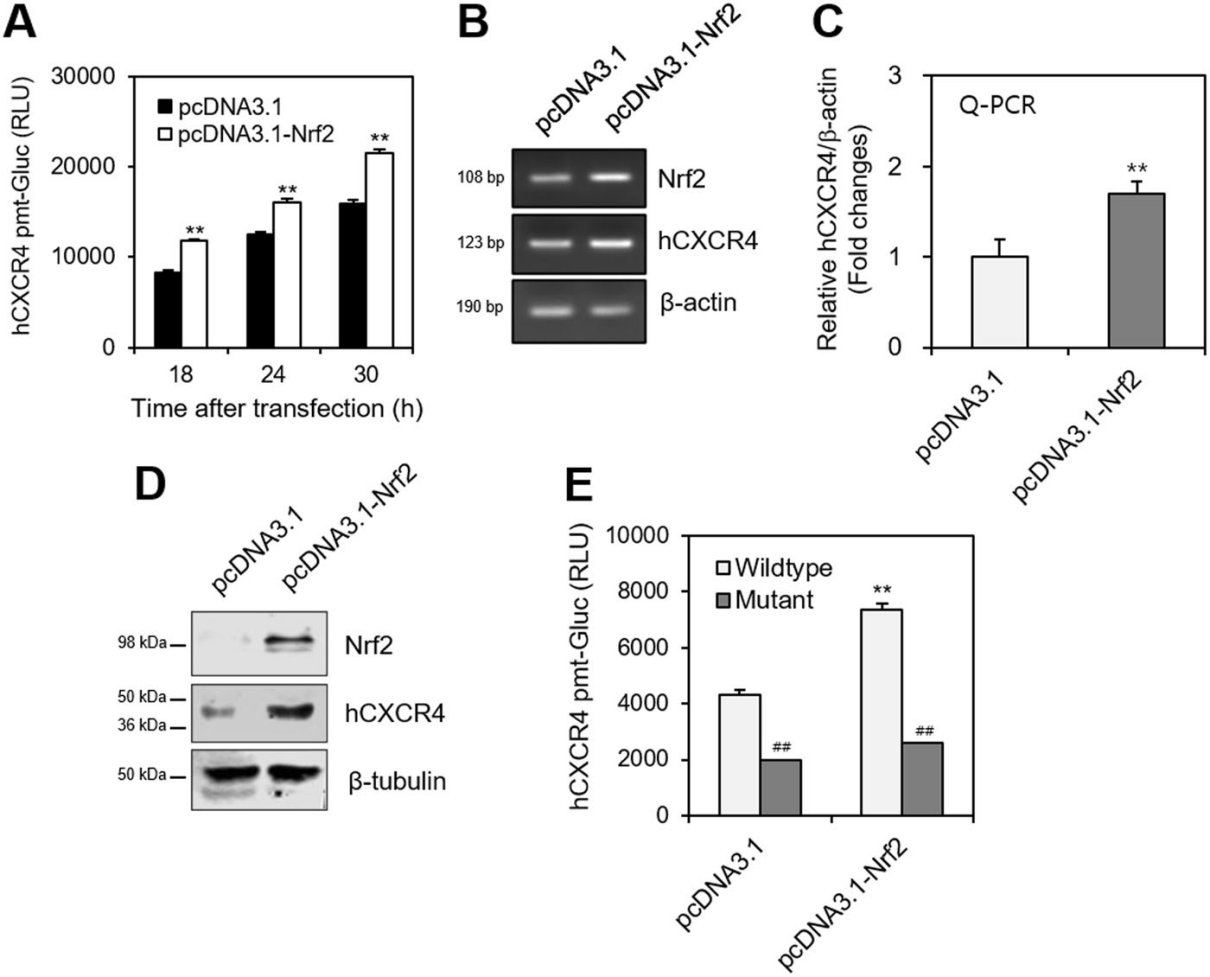
hCXCR4 expression was increased by Nrf2 overexpression. **A**–**D** WiL2-NS cells were co-transfected with pEZX-PG02-hCXCR4-gaussia luciferase (Gluc) plasmid DNA with pcDNA3.1 control or pcDNA3.1-Nrf2 plasmids using Viafect™. 30 h after transfection, Gluc activity of hCXCR4 promoter (pmt) was measured by using luminometer (**A**). RNA was extracted by using Nucleozol. mRNA of hCXCR4 was measured by RT-PCR (**B**) or qPCR (**C**). Cell lysates were prepared and the protein level of each protein was measured by using western blot analysis (**D**). **E** WiL2-NS cells were co-transfected with wild or mutant type of pEZx-PG02-hCXCR4-Gluc promoter plasmid DNA with pcDNA3.1 control or pcDNA3.1-Nrf2 plasmids using Viafect™. 30 h after transfection, WiL2-NS cells were incubated under hypoxic condition and Gluc activity was measured by using luminometer. Each experiment was performed at least five times. Data in a bar graph represented the means ± SD. *p < 0.05, **p < 0.01; significantly different from pcDNA3.1-transfected control group. ^#^*p* < 0.05, ^##^*p* < 0.01; significantly different from control transfected with wildtype CXCR4 pmt plasmid (**A**, **C**, **E**).

To confirm the effect of Nrf2 on hCXCR4 expression, WiL2-NS cells were co-transfected with hCXCR4-Gluc and pcDNA3.1-Nrf2 plasmid DNA. hCXCR4-Gluc activity increased ~42%, ~28%, and ~35% at 18-, 24- and 30-h after transfection, respectively, in response to the pcDNA3.1-Nrf2 plasmid DNA compared to that in response to control pcDNA3.1 plasmid DNA (Fig. 5A). The hCXCR4 transcript level increased in response to pcDNA3.1-Nrf2, which was confirmed by RT-PCR (Fig. 5B). hCXCR4 expression increased ~45% after transfection with the pcDNA3.1-Nrf2 plasmid DNA as judged by qPCR (Fig. 5C). An increase in hCXCR4 protein level in response to pcDNA3.1-Nrf2 was confirmed by western blot analysis (Fig. 5D). To confirm the effect of Nrf2 on hCXCR4 expression, we prepared the deletion mutant of the hCXCR4 promoter. When WiL2-NS cells were co-transfected with wild-type or mutant-type hCXCR4-Gluc and pcDNA3.1-Nrf2 plasmid DNA, hCXCR4-Gluc activity was inhibited by deleting upstream of the hCXCR4 promoter (Fig. 5E). These data suggest that Nrf2 directly controls hCXCR4 expression.

**Fig. 6:**
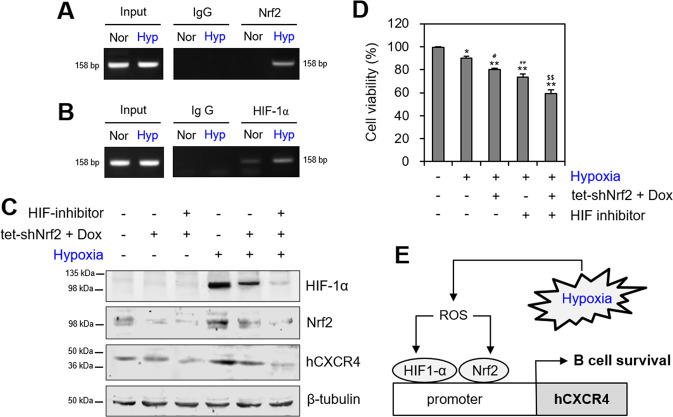
B cell viability was regulated by binding Nrf2 and HIF-1α on hCXCR4 promoter under hypoxic condition. **A**, **B** WiL2-NS cells were incubated under normoxic (Nor) or hypoxic (Hyp) condition and fixed with 10% formaldehyde. Their chromatin extracts were immunoprecipitated with anti-Nrf2 (**A**) or anti-HIF-1α (**B**) antibodies. DNA fragments were subjected to PCR analysis using primer sets spanning the promoter regions. Sequences for primer set were CTACATCTGATCAGTCTCCAG (forward) and AGCCCATTCAGGAGGTAA (reverse). Primer set corresponds to –718 to –561 bp including the second Nrf2 binding (–636 to –626 bp) on hCXCR4 promoter. **C**, **D** The infected shNrf2-positive control (tet-shNrf2, −Dox) and Nrf2-KD (tet-shNrf2, +Dox) WiL2-NS cells were incubated in the presence or the absence of HIF-1α inhibitor under hypoxic condition. Cell lysates were prepared and the protein level of each protein was measured by using western blot analysis (**C**). Cell viability was measured by using trypan blue exclusion assay. Each experiment was performed at least five times. Data in a bar graph represented the means ± SD. *p < 0.05, **p < 0.01; significantly different from control group under normoxic condition. ^#^*p* < 0.05, ^##^*p* < 0.01; significantly different from shNrf2-positive control (tet-shNrf2, −Dox) with untreatment of HIF inhibitor under hypoxic condition. ^$$^p < 0.01; significantly different from Nrf2-KD (tet-shNrf2, +Dox) or HIF inhibitor-treated group under hypoxic condition (**D**). **E** This is a schematic regulatory mechanism of B cell survival by binding Nrf2 and HIF-1α on hCXCR4 promoter under hypoxic condition. It suggests that hypoxia-induced ROS controls CXCR4 expression via the interaction of HIF-1α and Nrf2 on its promoter cooperatively.

Then, we predicted the Nrf2-binding sites in the hCXCR4 promoter sequence using the TRANSFEC (version 8.3) database (Supplementary Fig. S1, online). To examine the interaction between Nrf2 and the hCXCR4 promoter, we performed a ChIP assay using an anti-Nrf2 antibody. As shown in Fig. 6A, Nrf2 bound to the hCXCR4 promoter under hypoxic conditions. HIF-1α also bound significantly to the hCXCR4 promoter under hypoxic conditions (Fig. 6B). However, while no binding site for HIF-1α was predicted in the hCXCR4 promoter sequence using the TRANSFEC (version 8.3) database, the sequence (−650 to −644, GCACRTG) on upstream of the second Nrf2-binding site (−636 to −626, TGATGCTGTGA) was partially matched to HIF-1α-binding site (Supplementary Fig. S1, online). Then, hCXCR4 expression was cooperatively inhibited by treatment with the HIF inhibitor and Nrf2-KD (tet-shNrf2, +Dox) in WiL2-NS cells under hypoxic conditions (Fig. 6C). Cell viability was attenuated synergistically by treatment with the HIF inhibitor in Nrf2-KD cells under hypoxic conditions (Fig. 6D). These results suggest that hypoxia-induced ROS control CXCR4 expression through the cooperative interaction between HIF-1α and Nrf2 on its promoter. Through this molecular mechanism, CXCR4 regulates B cell viability under hypoxic conditions (Fig. 6E).

## Discussion

CXCR4 is a specific receptor for SDF-1 that control B cells at multiple stages of development^39^. CXCL12 is a homeostatic chemokine that signals via CXCR4^48^ and plays an important role in the development, hematopoiesis, and organization of the immune system^49^. CXCL12 binding to CXCR4 stimulates various signal transduction pathways that regulate intracellular chemotaxis, calcium flux, transcription, and cell survival^50^. Hypoxia and the HIF signaling pathway play important roles in B cell function and development, which are crucial for B cell survival, proliferation, and cytokine production^16^. Cancer treatment requires B cells, but inhibition is required in the case of autoimmune diseases^18,19^. Therefore, determining the factors that control B cell survival is critical for treating hypoxia-related diseases. Here, we investigated whether B cell viability is regulated by CXCR4 via induction of HIF-1α and Nrf2 under hypoxic conditions in WiL2-NS human B cells.

Cell viability decreased under hypoxic conditions by inhibiting CXCR4 expression in WiL2-NS cells. Also, hypoxia increased HIF-1α and Nrf2 expressions (Fig. 1). CXCR4 expression in WiL2-NS cells was inhibited by treatment with the HIF inhibitor, which also inhibited Nrf2 expression (Fig. 2). ROS production increased the expression of HIF-1α, Nrf2, and CXCR4 under hypoxic conditions, which decreased in response to NAC (Fig. 3). CXCR4 expression was inhibited by Nrf2 KD (Fig. 4), and increased by Nrf2 overexpression (Fig. 5). HIF-1α was also affected by Nrf2 KD (Fig. 4). CXCR4 expression increased by the binding of HIF-1α and Nrf2 to the CXCR4 promoter. B cell viability was cooperatively attenuated by Nrf2 KD and by treatment with the HIF inhibitor (Fig. 6). These results suggest that HIF-1α and Nrf2 cooperatively regulate CXCR4 expression under hypoxic conditions. It also suggests that hypoxia-induced CXCR4 protects B cells.

CXCR4 is a receptor that selectively binds SDF-1 known as CXCL12^30,31^, and CXCR4 is related to HIF-1α, which facilitates cancer cell survival^39^ through binding with its CXCL12 ligands^48,50^. It could explain the regulation of CXCR4 expression under hypoxic condition. Hypoxia-induced CXCR4 could bind more CXCL12 molecules and stimulates intracellular signaling pathways. Then, it is expected the possibility that there is a positive feedback mechanism by which CXCR4 regulates Nrf2 and HIF-1α expressions. It will be required to define the possible mechanism on B cell response by further study including other specific cells to release CXCL12.

In the meanwhile, if there is a positive feedback mechanism by binding CXCL12 to CXCR4, it is considered that the decrease in survival rate caused by inhibition of CXCR4 under hypoxia is due to the disruption of the antioxidant defense system brought by Nrf2. So, it might not rule out that CXCR4 and HIF-1α regulate cell survival independently under hypoxic condition.

It is also possible for CXCR4 to regulate B cell survival through the secretion of CXCL12 from WiL2-NS cells under hypoxic condition. However, CXCL12 is mainly released by cancer-associated fibroblasts (CAFs), macrophages^51^, and bone marrow stromal cells (BMSCs)^52^. B cells are recruited to stroma cells through CXCR4–CXCL12 interaction. Then, it is impossible for B cells to release CXCL12 and be influenced by its autocrine effect under hypoxic condition.

CXCR4-targeted therapeutic approaches are being evaluated in preclinical studies to treat various diseases, including cancers^49,53^. Inhibiting CXCR4 inhibits tumor growth, reduces lung metastasis, and improves survival after sorafenib treatment^54^. Our data show that CXCR4 also plays a role in controlling B cell survival under hypoxic conditions, suggesting that CXCR4 could be a pivotal molecule regulating B cells in the tumor microenvironment. In contrast, CXCR4 enhanced B-1a cell migration to bone marrow, which produce IgM antibodies during health and disease^55^. Thus, controlling molecular changes under hypoxic conditions is a therapeutic strategy for treating cancer and inflammatory disease^15^. Our data show that HIF-1α and Nrf2 may protect B cells against hypoxia-induced ROS by increasing CXCR4. The findings suggest that the strategy to control CXCR4 expression could differ depending on the cells targeting different types of diseases.

Hypoxia is a hallmark of infected, inflamed, or damaged tissue^56,57^. The increase of ROS in response to hypoxia causes oxidative damage to cells^58^, which occurs in most disease conditions^23,25,59^. Mitochondrial ROS are engaged in transcriptional and translational regulation of HIF-1α by inhibiting PHD^24^, particularly through the ERK and PI3K/AKT pathways^23^. The von Hippel–Lindau tumor suppressor protein pVHL negatively regulates CXCR4 expression owing to its capacity to degrade HIF under normoxic conditions^36^. Peroxisome proliferator-activated receptor gamma-dependent downregulation of CXCR4 in cancer cells slows the rate of metastasis^60^. So, controlling CXCR4 changes will be a useful strategy to treat various diseases.

Taken together, hypoxia-induced ROS may regulate CXCR4 expression through the cooperation with HIF-1α and Nrf2. Through this molecular mechanism, we suggest that B cell survival is regulated by hypoxia-induced CXCR4 expression, which could be a novel therapeutic target for hypoxia-associated diseases.

## Supplementary information

Supplementary figure 1
