# Supplementary material for: CXCR4 promotes B cell viability by the cooperation of nuclear factor (erythroid-derived 2)-like 2 and hypoxia-inducible factor-1α under hypoxic conditions
Source: Cell Death Dis. 2021 Mar 26;12(4):330. doi: 10.1038/s41419-021-03615-w (PMC7998033; doi:10.1038/s41419-021-03615-w)
Supplement: Supplementary file 1 — Supplementary figure 1 [file 41419_2021_3615_MOESM1_ESM.docx]

**CXCR4 promotes B cell viability by cooperation of nuclear factor (erythroid-derived2)-like 2 and hypoxia-inducible factor-1α under hypoxia condition**

Ju-Won Jang, Pham Xuan Thuy, Jae-Wook Lee and Eun-Yi Moon *

*^a^Department of Bioscience and Biotechnology, Sejong University, Seoul 05006, Republic of Korea*

Running title: CXCR4 promotes B cell viability by cooperation of HIF-1α and Nrf2

*Corresponding author

Eun-Yi Moon, Department of Bioscience and Biotechnology, Sejong University, 209 Neungdong-ro Kwangjin-gu, Seoul 05006, Republic of Korea,

Tel: +82 2 3408 3768; Fax: +82 2 466 8768.

E-mail address: [eunyimoon@sejong.ac.kr](mailto:eunyimoon@sejong.ac.kr) (E.Y. Moon)

**Supplementary Figure S1.** Schematic figures and sequences of pre-designed hCXCR4 (NM_003467) promoter. (A) hCXCR4 promoter (HPRM44928) was 1,309 bp (-1,328 ~ -35) upstream from starting codon, ATG, of coding sequence (CDS) for hCXCR4 transcription. hCXCR4 promoter covers 1,233 bp upstream and 60 bp downstream from transcription starting site (TSS). (B) Sequence of hCXCR4 promoter includes four Nrf2 binding sites (black) and HIF-1α binding site (italic grey underlined) which are predicted by using TRANSFEC (version 8.3) database, TSS (black bold), and nucleotides (grey underlined) to design primer sets for chromatin immunoprecipitation. Translation starting codon (italic black bold underlined) is shown in between -34 and 13 bp (italic grey bold underlined).
